# Aerosol Absorption: Progress Towards Global and Regional Constraints

**DOI:** 10.1007/s40641-018-0091-4

**Published:** 2018-04-03

**Authors:** Bjørn H. Samset, Camilla W. Stjern, Elisabeth Andrews, Ralph A. Kahn, Gunnar Myhre, Michael Schulz, Gregory L. Schuster

**Affiliations:** 1 CICERO Center for International Climate Research, Oslo, Norway; 2 CIRES, University of Colorado, Boulder, CO USA; 3 Earth Sciences Division, NASA Goddard Space Flight Center, Greenbelt, MD 20771 USA; 4 Norwegian Meteorological Institute, Oslo, Norway; 5 NASA Langley Research Center, Hampton, VA USA

**Keywords:** Aerosols, Absorption, Black carbon, Brown carbon, Mineral dust, Climate

## Abstract

**Purpose of Review:**

Some aerosols absorb solar radiation, altering cloud properties, atmospheric stability and circulation dynamics, and the water cycle. Here we review recent progress towards global and regional constraints on aerosol absorption from observations and modeling, considering physical properties and combined approaches crucial for understanding the total (natural and anthropogenic) influences of aerosols on the climate.

**Recent Findings:**

We emphasize developments in black carbon absorption alteration due to coating and ageing, brown carbon characterization, dust composition, absorbing aerosol above cloud, source modeling and size distributions, and validation of high-resolution modeling against a range of observations.

**Summary:**

Both observations and modeling of total aerosol absorption, absorbing aerosol optical depths and single scattering albedo, as well as the vertical distribution of atmospheric absorption, still suffer from uncertainties and unknowns significant for climate applications. We offer a roadmap of developments needed to bring the field substantially forward.

**Electronic supplementary material:**

The online version of this article (10.1007/s40641-018-0091-4) contains supplementary material, which is available to authorized users.

## Introduction

Improving constraints on aerosol absorption of light is a key challenge for current atmospheric research. Although it has long been known that some aerosol species perturb the energy balance of the atmospheric column through shortwave absorption, affecting radiative forcing, cloud formation, precipitation, and more, the magnitude of these effects has proven difficult to pin down. A 2009 review paper [1] concluded that although progress was rapid, “there is need for much additional work in characterizing aerosol light absorption in the atmosphere and its effects on radiative forcing and visibility.” Despite a subsequent decade of active research and considerable progress, this statement is still generally true. However, recent developments and suggestions for future observations show great promise, indicating that better constraints on aerosol absorption may be possible in the near future.

Aerosols affect the climate system by intercepting incoming shortwave radiation. Although all aerosols act as scatterers of radiation, reducing surface irradiance, some species also absorb, effectively adding a positive energy term to the atmospheric radiative balance. The main absorbing aerosol species are black carbon (BC) [2], mineral dust [3, 4], and the absorbing component of organic aerosols [5, 6], recently termed brown carbon (BrC). Conceptually, the net shortwave aerosol absorption, usually quantified through the absorbing aerosol optical depth (AAOD), can therefore be thought of as the sum of the contributions of these three separate species, integrated over the atmospheric column. Observationally, however, such clear distinction into separate aerosol categories is usually not possible, because of mixing of aerosol species. This makes validation of model predictions challenging.

Further, the aerosol types—however they are defined—differ significantly in emission volumes, locations, and seasonality, in their transport and residence times in the atmosphere, and in the spectral dependence of their absorptive (and other optical) properties. Aerosol loading varies significantly with geographical location and season, and depends upon annually varying conditions such as meteorology, soil moisture, and the intensity of fire seasons. Aerosol absorption is primarily retrieved via remote sensing (e.g., satellites and ground-based sunphotometers) or measured via in situ instruments at long-term surface stations and during aircraft field campaigns. Although a wealth of observational data exists, these data are still far from sufficient to provide global and annual coverage of such diverse and rapidly varying quantities. Present efforts therefore focus on combining model calculations with observations through gap filling, assimilation techniques, and reanalysis.

In the present review, we define “constraint” as reasonable agreement between observations and theoretical or model-based estimates, combined with a quantification of the agreement and some understanding of why the two agree. We think that a reasonable agreement is achieved if global anthropogenic aerosol forcing due to aerosol radiation interactions from absorbing components could be estimated within 0.1 W m^−2^. To break the problem down, we first cover recent advances in modeling and observational constraints on the physical properties of individual aerosol species. Topics of particular interest are absorption changes from ageing (coating and fractal collapse) of BC, and improved understanding of BC particle morphology and mixing state. For brown carbon, the main challenges are the spectral absorption dependence and ageing properties, as well as a clear distinction from BC and dust. For mineral dust, important issues are the uncertainties associated with composition, size, and source distribution (and changes in these properties during transport), as well as modeled source terms. Next, we cover advances in observations via remote sensing and in situ measurements, and recent reanalysis results. We also discuss a range of outstanding issues and how they currently preclude sufficient constraints on aerosol absorption to guide global or regional climate models for climate forcing applications. A roadmap towards improved constraints on aerosol absorption, as defined above, is given in Table 1. The recommendations we set out are drawn from the discussions of recent literature in the coming sections, and from discussions at topical workshops such as the annual AeroCom/AeroSAT meeting. A summary of the major topics in recent literature, including some key publications, is also provided in Supplementary Table 1.

**Table 1:** A roadmap towards improved constraints on aerosol absorption. A constraint is here defined as agreement between observational and theoretical/modeling estimates, and some understanding of why they agree. The table lists developments that, in our view, are key to bringing the field forward

Basic/immediate recommendations	
What	Improved dialogue between aerosol observational and modeling communities
How	Continued focus on dedicated meetings, such as the annual AeroCom/AeroSAT workshops
What	Use consistent terminology for BC, in both observational and model studies
How	Adhere to recommendations in Petzold et al. 2013, clearly define fresh/collapsed and young/old in optical parameter studies, avoid confusion with brown carbon
What	Rigorous treatment of BrC, in observations and models
How	Extend definition, discuss as part of spectrum of carbonaceous combustion products. Include in broader set of climate models. Develop emission estimates
What	Consistent usage of AERONET observations
How	Adherence to quality flags, improved understanding of the impact of retrieval assumptions and treatment of the representativeness of site locations, closure studies using airborne in situ measurements and sun photometers, bias correction for cloudy and low AOD days
Developments/longer term recommendations	
What	Improve microphysical treatment of aerosols in climate models
How	Include microphysics packages, multiple size modes, constrained physical properties based on observations. Rapid adoption of observational constraints, e.g., of optical properties
What	Improve satellite remote sensing sensitivity to absorbing-aerosol amount and type
How	Develop global, broad-swath, UV to NIR multi-spectral, multi-angle, and polarization imaging capabilities
What	Develop climatology of average aerosol optical properties, geographically, vertically, and seasonally resolved
How	Systematic aircraft measurements, coordinated as appropriate with ground based and satellite observations, and used as further constraint for climate models
What	Constrain absorption from aerosols above clouds
How	Develop/improve satellite retrievals, aircraft observation programs, and dedicated model studies
What	Constrain BC emissions, transport, ageing, geographical, and vertical distributions
How	Targeted in situ aircraft and ground sampling programs, in collaboration with modeling groups, explore constraints from measured long-term absorption trends in different regions, document all relevant aspects of modeled life cycles of BC, BrC, and dust
What	Heighten focus on the role of dust
How	Measure and model optical properties of broader set of dust types, especially coarse-mode dust. Implement in retrieval algorithms and transport models

As the present format is not suited to review the underlying theory or core experimental and numerical techniques, we refer the reader to the reviews and summaries of Moosmüller et al. [1], Bergstrom et al. [7], Petzold et al. [8], Lack et al. [9], and Stier et al. [10].

## Motivation

The present lack of good constraints on aerosol absorption can significantly affect estimates of aerosol climate impact. As an example, we consider the recent Phase 2 of the AeroCom multi-model initiative. There, modelers simulated aerosol loading and radiative impacts for the same meteorological year (2006) using identical emissions (year 2000, [11]). They reported a multi-model annual mean total AAOD at 550 nm of 0.0042 ± 0.0019 (1 std.dev.), with a min-max range of [0.0021, 0.0076] (see Myhre et al. [12]). Anthropogenic AAOD at 550 nm was reported as 0.0015 ± 0.0007, with a global relative standard deviation of ~ 50%. (See further discussion of AeroCom Phase 2 below, and Fig. 2.)

To test the implications of this uncertainty for climate simulations, we modified the optical properties of BC in a recent climate model (CESM 1.2 using CAM5 [13] with year 2000 conditions and fixed sea-surface temperatures), by a quantity sufficient to increase the total annual mean AAOD by approximately one AeroCom Phase II standard deviation (AeroCom multi-model historical AAOD: 0.0015 ± 0.0015; our baseline was 0.0030, perturbed to 0.0043). By comparing the simulated perturbed setup to one using the default CAM5 optical parameters for years 3–30, we find an instantaneous, top-of-atmosphere effective radiative forcing of up to 3 W m^−2^ over BC emission hotspots (global mean: 0.2 W m^−2^, which is comparable to the historical aerosol RF of the direct effect in AeroCom Phase II: − 0.27 ± 0.15 W m^−2^). Calculation of effective radiative forcing (ERF) followed Forster et al. [14]. Even with fixed sea-surface temperatures, this forcing induces land surface temperature changes of up to + 0.5 K over midlatitude regions, and up to + 1 K over high albedo surfaces such as Greenland—far from emission regions. Notable changes are also simulated for precipitation and cloud fractions, as parts of the response to the forcing. Although this setup is idealized, and results would differ if mineral dust or brown carbon was perturbed instead of BC, it indicates the magnitude of inter-model differences possible within the present spread of predicted AAOD. Recently, it has also been shown that BC, as a significant contributor to atmospheric absorption, is likely a main driver of inter-model differences in precipitation predictions ([15, 16]). In our simplified simulation mentioned above, atmospheric column energy absorption also changed by up to 5 W m^−2^ in regions with high BC emissions, likely affecting atmospheric stability and precipitation rates. Clearly, better constraints on AAOD would aid the development of coupled climate models.

Another notable example of the importance and understanding of aerosol absorption is the use of AAOD constraints from AErosol RObotic NETwork (AERONET) stations (to be discussed below) in a recent assessment of the climate impacts of BC emissions [2]. Model results were scaled to match AERONET, resulting in a marked increase in estimated global BC RF, as it was calculated using model-estimated forcing efficiencies per unit AAOD. Similar approaches are often used with various underlying assumptions about aerosol composition and corrections for regional or near-source bias.

In response to this challenge, there is considerable activity in the aerosol science community to improve knowledge of aerosol absorption. As an indication, Supplementary Fig. 1 shows the recent evolution in the number of publications within the earth sciences dealing with aerosol absorption in general and in combination with one of the major aerosol species. (For information on how the selection was made, see the figure caption.) The field has seen a doubling in the number of publications over a 15-year period. Topically, the literature is moving increasingly from discussing “aerosols” in general, to focusing on the three main absorbing species. Brown carbon has seen a sharp rise in interest after 2010, and by 2016 had almost as many publications as the longer standing topic of dust absorption. Interest in black carbon absorption is also at a record high.

## Species-Based Advances and Perspectives

**Fig. 1:**
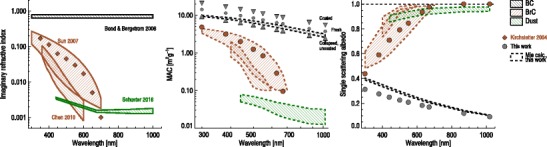
Optical parameters for the three main absorbing aerosol types, with values guided by recent literature. Left: imaginary refractive index (*k*) as function of wavelength. Values are from [17] (BC), [6, 18, 19] (BrC), and [20] (dust). The dust values represent the range of an AeroNet-derived climatology. In the middle and right panels, the *k* values have been used as input to Mie theory calculations, to yield consistent values for MAC and SSA. Mie calculation results are shown with dashed borders. For BC and BrC, size distributions were taken from standard calculations of accumulation type aerosols [21] (Radius (NMR) and sigma (GSD) of 0.04 μm and 1.5 for BC, and 0.05 μm and 2.0 for BrC). For mineral dust, the observed size distribution from the DABEX aerosol campaign was taken as input [22]. Aerosol densities applied in the Mie calculations were 1.2, 1.8, and 2.6 g cm^−3^, for BrC, BC, and dust, respectively. Freshly emitted BC is often composed of aggregates, sometimes thinly coated, with a representative MAC of 7.5 m^2^ g^-1^. Using Mie theory with the recommended refractive indices, size distributions and density are therefore inconsistent with observed MAC of freshly emitted BC (see text for further discussion). To illustrate the optical properties of common, freshly emitted BC, we show additional MAC and SSA values (gray circles) where the Mie calculations have been scaled to achieve the recommended MAC of 7.5 m^2^ g^−1^ at 550 nm. In the MAC panel, we also indicate the range of values found in the literature for coated BC, and collapsed, uncoated BC (see main text and Supplementary Table 2)

One path towards improved constraints on absorption is to treat the aerosol population as a collection of individual species—notably BC, BrC, and mineral dust. In this section, we discuss known issues and recent key insights for these species individually. Both physical and optical properties, and their implementation in present climate models, are treated. We note that although separation into the categories “BC”, “BrC,” and “mineral dust” makes sense in models, where separate sources and collective properties can be fully specified, such distinctions will always be idealized. E.g., BC and BrC both belong to the spectrum of carbonaceous combustion products, which can have a wide range of properties. However, there is broad agreement that, in practice, there exist general categories of absorbing aerosols that have distinct physical properties. Understanding and constraining these properties are crucial first steps towards also constraining total aerosol absorption. Figure 1 illustrates these differences, sketching the present knowledge of imaginary refractive index wavelength dependence, mass absorption coefficient (MAC), and single scattering albedo (SSA) for BC, BrC, and dust. The bands indicate, but do not exhaustively cover, values that appear in the literature. Also, Supplementary Table 1 provides a summary of the issues to be discussed below and in the next section. For a discussion on the emergent power law behavior of aerosol absorption as function of wavelength, quantified through the Absorption Ångström Exponent (AAE), see, e.g., Andersson [23].

### Black Carbon

BC is a collective term for strongly light-absorbing, carbonaceous aerosols, arising from incomplete combustion of fossil fuels, biofuels, and biomass. BC is one of the aerosol species that contributes the most to atmospheric absorption under present-day conditions. Different sources and combustion temperatures give rise to variations in particle structure and shape [5, 24]. Further changes in morphology and hygroscopicity occur as BC particles age, and, in the process, the particles can grow and mix with other atmospheric constituents, inducing spatial and temporal variations in their optical properties.

#### Definition and Optical Properties

There is still significant ambiguity as to the definition of BC in the scientific literature. However, most recent studies adhere to the definition of Petzold et al. [8] of BC as “an ideally light-absorbing substance composed of carbon,” and the more detailed definition by Moosmüller et al. [1] as “carbonaceous material with a deep black appearance, which is caused by a significant, nonzero imaginary part . . . of the refractive index that is wavelength independent over the visible and near-visible spectral regions.” The latter property of BC is illustrated in Fig. 1, which shows that the AAE is essentially unity. We note, however, that this might not be the case for collapsed BC aggregates [20, 25, 26] or internally mixed BC [20, 26], and should be viewed as a simplifying assumption only. E.g. Liu et al. [27] recently argued that the BC AAE generally is slightly lower than unity, based on advanced optical modeling and realistic particle geometries using fractal aggregates. However, we note that one can not use AAE alone to separate carbonaceous aerosols from dust, since AAE is also affected by particle size [26]. Consequently, the competing effects of large particle size driving AAE downward and hematite concentration driving AAE upward result in pure dust AAEs that can vary from less than 0 to greater than 3 [20].

Since BC never occurs in the atmosphere as pure carbonaceous matter [8], its optical properties are highly dependent on particle age, and on atmospheric conditions including the relative humidity and the availability of gaseous precursors for coating. This challenges both empirical definitions of BC for use in interpreting observations, and the ability of models to estimate global average absorption by BC. A 2006 review by Bond, Bergstrom [17] investigated the current theoretical understanding as well as observations, and proposed a representative MAC value of 7.5 m^2^ g^−1^ (at 550 nm) for freshly generated BC. They suggested a range in MAC values from about 5 m^2^ g^−1^ for collapsed but uncoated BC, up to about 11 m^2^ g^−1^ for aged and coated BC (see Fig. 1). This range is consistent with more recent observations, in spite of a relatively large spread that reflects differences in measuring techniques, the type of airmass measured, mixing state, and proportion of BC in the aerosols [28–30]. As examples, Cui et al. [31] report average MAC values around 10 m^2^ g^−1^ (at 678 nm) for a site in rural North China; Ram, Sarin [32] find values between 6 and 14 m^2^ g^−1^ (at 678 nm) at different sites in India, whereas a lower value of around 6 m^2^ g^−1^ (at 522 nm) is found for the Arctic [33]. Zanatta et al. [34] find an annual mean MAC value of 10 m^2^ g^−1^ (at 637 nm) to be representative of the mixed boundary layer at European background sites.

#### Ageing, Coating and Absorption Enhancement

The range of reported MAC values as discussed above reflects that as BC mixes with other aerosol species, it can become coated, which enhances its absorptive properties, while the fresh fractal aggregates can collapse, which will reduce the enhancement. These processes are often collectively termed “BC ageing,” even if sometimes only one of the two is implied. Laboratory experiments (e.g., [35–37]) find combined enhancement factors (E_abs_, defined as the ratio of absorption by aged BC relative to freshly emitted aerosol) of similar magnitude to those obtained in earlier measurements [17], but recent observational estimates show a large spread. Bond, Bergstrom [17] recommended a global enhancement factor of 1.5, a number which has been widely used in climate models. This means that assumed MAC values of fresh BC 7.5 m^2^ g^−1^ would correspond to MAC values of 11 m^2^ g^−1^ of the aged BC.

Several other studies have investigated E_abs_ through observations, finding values raging from 1.0 to 3.0. See Supplementary Table 2 (ST2) for a (non-exhaustive) overview of recent observationally based estimates. Some of the differences between estimates of E_abs_ can be attributed to the use of different instrumentation and methodologies (e.g., [38]), variations in the fraction of BC that is internally mixed (e.g., [25]) or dominance of different source contributions to the BC measured (e.g., [39]). A further source of confusion is the difference in baseline, from which the enhancement is calculated. Although the recommended E_abs_ of 1.5 represents absorption enhancement from freshly emitted to fully aged BC, some studies give E_abs_ values for samples dominated by fresh local sources, relative to uncoated pure BC (e.g., [35, 40]). These E_abs_ measurements have been categorized as E_abs,fresh_ in ST2, and typically show relatively small values. A few studies report enhancement between fresh and aged BC (E_abs,aged_ in ST2), and these correspond reasonably to the value of 1.5. Yet other studies give the enhancement from pure uncoated BC to fully aged, producing in some cases very high E_abs_ values (e.g., [31, 41]).

In climate models, E_abs_ is calculated based on mixing state assumptions, or its value is simply set to a constant number (typically 1.5, as recommended in [17]). There is a large spread in the way models treat BC, and many models even assume that all BC are externally mixed. This produces substantial differences in modeled MAC values. For instance, although Boucher et al. [42] use the indications from observations that MAC values should be somewhere in the vicinity of 10 m^2^ g^−1^ (at 550 nm) on average, a multi-model study by Stjern et al. [16] shows a model mean MAC value of 6.3 m^2^ g^−1^ (at 550 nm), but with individual model values ranging from 3 to 10 m^2^ g^−1^. These differences contribute to the large inter-model spread in BC absorption estimates.

New attempts to improve the parameterization of absorption enhancement in models are emerging in the literature. For instance, Fierce et al. [43] compare absorption enhancement in BC populations where the mass fraction of each aerosol component is assumed to be the same for all BC containing particles, to the enhancement found when using a model that resolves individual particles. They find that the latter approach yields an E_abs_ of 1 to 1.5 at low relative humidity, consistent with ambient observations, whereas if a population-averaged composition is assumed across all BC-containing particles, absorption is strongly overestimated (E_abs_ > 2).

Liu et al. [44] use a generalized hybrid model approach for estimating scattering and absorption enhancements based on laboratory and atmospheric observations. They developed a method for determining when the BC is significantly enhanced by non-BC, based on the relative mass ratio of non-BC to BC in a single particle, which was already known to be important for E_abs_ [20, 26]. Where the relative mass ratio is less than 1.5 (e.g., coatings typical of fresh traffic sources), BC is best represented as having little or no bulk absorption enhancement (E_abs_ = 1.0–1.4). For ratios greater than 3 (typical of aged biomass-burning emissions), BC is best described assuming optical lensing leading to an absorption enhancement (E_abs_ > 1.6). (We note that the term “lensing” is a geometric optics concept that has little meaning for sub-wavelength particle sizes, as is typical for combustion aerosols. However, we acknowledge that the term is widely used by the community and has a clear definition.) These developments are steps toward improving the representation of BC absorption in models.

#### Residence Time, Vertical Profiles, and Emission Inventories

Although absorption enhancements due to ageing is a key topic in recent BC literature, a number of other poorly constrained factors also contribute to the present spread in model results. As the absorption of an aerosol layer depends upon the albedo of the underlying surface, both the average residence time of atmospheric BC and its vertical concentration profile will influence absorption estimates and environmental impacts. For example, the HIPPO flight campaigns over the Pacific highlight a tendency in models to overestimate BC concentrations aloft [45], consistent with a general overestimate of residence time in the models [46]. Uncertainty in BC emission inventories is a further factor. Estimated global, annual mean emissions range from 4 to 14 Tg year^−1^ [2, 47, 48]. Recently, Wang et al. [49] used a high resolution emission inventory over Asia, in combination with AERONET data, to further constrain BC AAOD through a Bayesian framework. They found significantly improved agreement when high resolution emissions were used, but also strong sensitivity to assumptions regarding BC ageing and transport. We discuss this study further below.

Recent developments in instrumentation are covered in subsequent sections on BrC and measurements.

### Brown Carbon

BrC is a catch-all term for the absorbing components of organic carbon aerosols (OA), which unlike BC, absorbs strongly at short wavelengths, but less toward the near-infrared part of the spectrum. Figure 1 shows the presently weak constraints on BrC optical properties, and the broad range of values that might actually apply under different circumstances. For a recent overview, see, e.g., Feng et al. [50], and references therein.

BrC is thought to be emitted primarily from biomass and biofuel burning [51–53], but it has also been seen in incomplete combustion of fossil fuels [54] and as a secondary organic aerosol [55–57].

A particular form of brown carbon that has received much recent attention is called tarballs; amorphous carbon spheres with mode diameters that range from about 100 to 300 nm, but individual particle sizes ranging from ∼ 25 nm to over 1 μm [58–60].

These near-perfect spheres are formed by gas-to-particle conversion during periods of high PM concentrations. They are larger than soot spherules, and lack the graphitic plate-like structure of soot. Tarballs are similar in composition to other organics [61, 62], but they are hydrophobic at relative humidities (RH) less than about 83% [58]. They can become soluble and weakly hygroscopic when RH > 83%, but they do not deliquesce. Consequently, the spheres retain their shape and remain largely isolated, although aggregation is sometimes observed [58].

Tarballs occur in almost all smoke from biomass burning, independent of fuel type [60]. Biofuel burning can also create tarballs, and Alexander et al. [59] found them to be ubiquitous in the East Asian Pacific outflow. The proportion of tarball particles in smoke increases as the smoke ages, and can dominate the carbonaceous aerosol contribution within minutes; because it takes time for the gas-to-particle conversion process to form tarballs, and other aerosol quantities are simultaneously decreasing with age [60].

Tarballs are more strongly absorbing than other forms of brown carbon when measured in isolation. Alexander et al. [59] used electron energy-loss spectrums in transmission electron microscope images to estimate a mass absorption efficiency of 3.6 to 4.1 m^2^ g^−1^ at 550 nm for individual tarballs. This is significantly greater than the values that we present for brown carbon in Fig. 1, and significantly less than the value of 7.5 m^2^ g^−1^ recommended by Bond, Bergstrom [17] for soot carbon. Alexander et al. [59] determined an imaginary refractive index of k(550) = 0.27 for the 28 tarballs that they analyzed, which is also intermediate of humic-like brown carbon and soot carbon. Aerosol transport models at present generally do not include tarballs as a separate species from brown carbon, but that is appropriate given that tarballs, brown carbon, and organic carbon are not separatable by mass with current measurement techniques.

#### Distinguishing BrC from BC

Although the differences in spectral absorption between BC and BrC are profound, making it possible to distinguish the two observationally, considerable effort is still required to characterize the composition and microphysical properties of BrC. This includes its absorption cross section and subsequent impact on global aerosol radiative forcing. Also, we note that modeling studies still sometimes use the term “black carbon” to include all non-dust absorbing aerosols, despite the vast differences in particle properties that are especially important for climate forcing calculations. Such unclear terminology can create significant issues when interpreting model results [5, 8, 63].

Recent studies indicate a positive, global BrC RF ranging from 0.1 to 0.6 W m^−2^, based on remote sensing [49, 64, 65] and transport modeling [50, 52, 66–70]. The spread in results is likely affected by differing assumptions on BrC composition, optical properties, emissions, and transport. The microphysical properties of absorbing OA are also likely to be highly sensitive to particle formation processes, and to the details of their evolution in the atmosphere.

In an attempt to constrain the relative absorption contributions from BrC and BC from combustion emissions under controlled conditions, Pokhrel et al. [38] recently reported measurements for a wide range of biomass fuels. At 405 nm, they find that BrC contributes of up to 92% of total absorption, but this fraction declined to 58% at 532 nm. Critically, they also report that the BrC component varies by a factor of two between analysis assumptions commonly made in the literature. Absorption enhancement due to lensing from coating, as discussed for BC above, provides a further complication, as fresh BC and BrC may be coated with partly absorbing organics. For example, Saleh et al. [52] found a strongly nonlinear interplay between absorption and lensing for organic aerosols, and Pokhrel et al. [38] note that results on lensing are highly method and model dependent. Further, it has been suggested that BrC may lose its absorbing properties as it ages, on relatively short timescales of hours to days. Recently, instrumentation has become available allowing quantification of this effect under field conditions [71–73]. However, to our knowledge it is not presently implemented in global models, and may lead to significant revisions in the partitioning of total absorption between BC and BrC at short wavelengths.

#### BrC Atmospheric Absorption

Accounting for BrC absorption in the atmosphere has also seen rapid development, mainly employing spectrally resolved observations from AERONET. Initially, AAE = 1 was often assumed for BC, and any nonlinearities subsequently ascribed to BrC (see, e.g., Olson et al. [54], and refs therein). Some studies refined this by grouping observational sites by region, and estimating optical parameters based on assumed single-source observations (e.g., [65, 74]). However, other authors have demonstrated that AAE does not contain enough information to unambiguously speciate the absorbing aerosols. See, in particular [26], and references therein. Briefly, AAE is affected by size; consequently the AAE of dust can hold a wide range of values (including less than 1), which in turn means that one can not use AAE alone to separate carbonaceous aerosols from dust.

Wang et al. [73] recently reported another approach that instead uses theoretical Mie calculations for BC, and no source assumptions. They find a global BrC absorption contribution of up to 40% of total carbonaceous aerosol absorption at 440 nm, and a mass absorption coefficient for OA (here defined as the total group of organic aerosols in the GEOS-Chem model) that positively correlates with the BC-to-OA mass ratio. Detailed analysis at two urban sites revealed no significant ageing effect on the MAC value, whereas it was found to decrease with a half-life of 1 day at a biomass burning site. This indicates, consistent with several other studies, that BrC properties cannot be taken as globally uniform.

Recently, some regional constraints on BrC absorption and vertically resolved abundances have become available from aircraft observations [70, 75, 76]. Zhang et al. [70] show that over the continental USA in May to June 2012, BrC was prevalent throughout the troposphere. As the climate responses to absorbing aerosols change with altitude [77], the authors suggest that high altitude BrC lofted from biomass burning events should be further studied. This was for instance done by Peterson et al. [78], who developed a satellite-based inventory for pyrocumulonimbus clouds, which can elevate smoke particles into the upper troposphere and even the lower stratosphere. Promisingly, they find good correspondence between combined BC and BrC absorption determined from merging aircraft observations with radiative transfer calculations, and that obtained from remote sensing results from Ozone Monitoring Instrument (OMI) at two wavelengths (see below).

#### BrC (and BC) Instrumentation

Recently, there has been great improvement in instrumentation for in situ observation, and separation, of atmospheric BC and BrC. Since the mid-2000s, many long-term monitoring sites and aircraft campaigns have used instruments such as the three-wavelength PSAP (Particle Soot Absorption Photometer) instrument, but due to its limited wavelength range (467, 530, 660 nm), spectral separation of BC and BrC is difficult. Seven-wavelength aethalometers, which are widely deployed at surface sites around the world, are better able to segregate BC and BrC [54, 79–81] due to their extended spectral range (from ultraviolet to near-infrared), but still have issues because in real situations the AAE of BC varies with particle size and mixing state. A serious potential limitation of filter-based absorption instruments such as PSAPs and Aethalometers is that by collecting the aerosol on a filter the particles’ physical and thus optical properties may be changed. Lack et al. [36] showed that PSAPs may overestimate absorption relative to photo-acoustic instruments, depending on the amount of organic aerosol present. Filter-based instruments at long-term monitoring sites are typically operated to measure at low RH (< 40%) (e.g., [82]); the drying required to achieve these RH levels can also affect the resulting absorption measurement, although the magnitude of this effect has not yet been quantified. The recent aircraft measurement papers cited above use a combination of Cavity Ring-Down (CRD) and Single Particle Soot Photometer (SP2) measurements. These instruments are much more sensitive to extinction or particle size, and can compute absorption from size distributions using assumptions about BC density and refractive index. However, they are difficult to operate unattended, precluding long-term, automated measurements. They also typically operate at non-ambient temperature and relative humidity conditions, as the sample air is brought into the aircraft and instrument. Upcoming campaigns will add photo-acoustic instruments, or the CAPS PM_ssa. Both the CAPS PM_ssa as well as some photo-acoustic instruments (e.g., the DMT PASS and PAX instruments;[83]) have the advantage of using the same sample volume to measure scattering and extinction to obtain SSA. This is a great improvement over previous in situ methods that required at least two instruments to determine SSA.

### Mineral Dust

Wind-blown mineral dust is thought to be the most abundant atmospheric aerosol by mass, at least in most global aerosol models [84, 85], and influences both shortwave and longwave radiation. Given its high abundance, absorption from dust can dominate that of black and brown carbon in some regions and seasons [86]. However, even though there is significant variability in the regional composition and spectral absorption of mineral dust (e.g., [87, 88]), models typically use a single dust “type” and ignore regional dependencies of the complex refractive index. Absorption of aeolian dust is mainly caused by the minerals hematite and goethite, but the refractive indices of these minerals are not well known despite their impact on derived forcing [89]. There is significant variability in the published refractive indices of hematite [20] and we know of only two studies that presents goethite refractive indices [90, 91]. Given the prevalence of goethite [87, 92, 93] and the important role that this mineral plays in altering the AAE of dust aerosols, more work is needed to characterize the refractive index of goethite and incorporate regional refractive index variability associated with the hematite/goethite ratio into global models.

Figure 1 indicates the range of optical properties attributed to mineral dust. As with BrC, it is usually taken to have an AAE significantly larger than 1 (see, e.g., [94]), although it can attain a very wide range of values depending on the actual ratio of minerals in the sample. Recently, Ridley et al. [95] used observations to constrain the dust AOD from the AeroCom Phase 2 model median of 0.023 (0.010 to 0.053) [96] to 0.030 (0.020 to 0.040), slightly increasing the AOD estimate and significantly reducing the spread. But many sources of model uncertainty and biases in dust absorption estimates remain, related to optical properties, in part because complex particle shapes are exceedingly difficult to model, but also because size distributions, shape, composition, and source terms are highly variable and poorly constrained by observations.

#### Loading and Size Distributions

Using satellite as well as ground-based measurements to constrain the global dust loading and its size distribution, Kok et al. [85] find that models overestimate emissions of fine dust and underestimate emissions of very coarse dust. Fine dust (D ≤ 2 μm) with sizes on the order of solar wavelengths produce the largest shortwave scattering impact per unit aerosol mass (so generally cooling), whereas larger particles (diameter similar to wavelength of terrestrial radiation) have the largest longwave absorbing effect (so warming), based upon results from one global model [97]. Compared to fine dust, larger dust particles have stronger absorption in the solar spectrum as well [85]. Therefore, the size bias in dust emissions means that models will tend to underestimate the solar absorption by dust, as found from satellite/lidar measurements over the global oceans in Lacagnina et al. [98]. Another bias, limiting constraints on both extinction and absorption from dust, may arise from the tendency of models to approximate dust aerosols as being spherical in shape. Kok et al. [85] compare the dust extinction efficiency of spherical dust to that of tri-axial ellipsoids [99], and find the model assumption of sphericity to underestimate dust extinction efficiencies by as much as 20–60% for dust larger than 1 μm. Non-spherical scattering models that can accommodate distributions of complex particle shapes and orientations with reasonable computational speed have not been developed thus far. Spherical or ellipsoid models used as basis for radiative flux calculations do not seem to work well for radiance-based satellite retrievals of dust AOD, e.g., from Multi-angle Imaging SpectroRadiometer (MISR) [100]. Further, there is considerable variation and uncertainty in the absorption properties of dust from different sources [4], and observational constraints are difficult to apply to climate models due to a lack of adequate optical models, particularly single-scattering phase functions, for coarse-mode non-spherical dust of all types (e.g., [100]). Some models do use spheroids, showing that treatment of non-spherical dust is possible [101]. The lack of optical characterization of goethite in the literature is critical since this is the most abundant form of iron oxide in dust [102], and the major light absorber in the shortwave spectrum [103].

#### Composition

Di Biagio et al. [104] performed in situ measurements of a number of different soils from eight different regions, and found that although the fraction of scattering by the different dust types varied little, there was great variation in the light absorption from region to region and also for various sources within regions, with significant correlations to mineralogical composition. They suggest that using regionally dependent refractive indices rather than generic values can yield significant improvements in modeling of dust radiative effects. Similarly, e.g., Engelbrecht et al. [105] measured size distributions and the spectral SSA of surface dust from 18 countries (including China, USA, Australia, and multiple countries in Africa). Although they reported a range of SSA values, at 550 nm almost all dust samples had SSA > 0.95.

### Multi-species, Model-Based Constraints

**Fig. 2:**
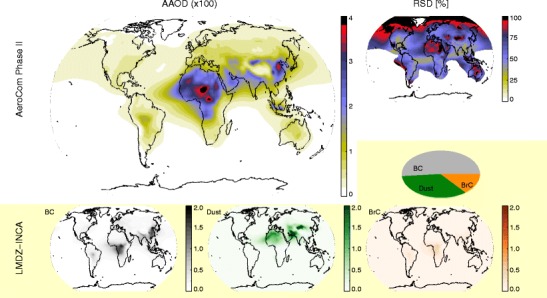
Top: total, annual mean aerosol absorption optical depth (AAOD) from AeroCom Phase II (multi-model mean), and the relative standard deviation (RSD) between the 16 participating models. All models used consistent anthropogenic emissions from year 2000, and year 2006 meteorology. However, biomass burning emissions and dust source parameterization varied between models. RSD is only plotted for bins with AAOD > 0.0015. Bottom: simulated AAOD due to BC (gray), dust (green), and BrC (brown) in a single model (LMDZ-INCA). The pie chart indicates the relative contributions of each species to the global AAOD of that particular model

Climate models try to combine the contributions to atmospheric absorption from several species into one estimate of the total global aerosol absorption, which can in turn be validated against observations. However, due in part to the issues raised above, inter-model differences in predicted AAOD are very large, even when using consistent emissions and nudged or prescribed meteorology. This issue was introduced in the Motivation section above, using the results from AeroCom Phase 2 [12]. Figure 2 further illustrates this, showing AAOD evaluated at 550 nm by the 16 global aerosol-climate models participating in that intercomparison. Some overall features are well captured by all models, including absorption by anthropogenic and biomass burning BC over Asia, Europe, Africa, and South America, dust from northern Africa and central Asia, and BrC from the major biomass burning regions for the few models that had included this component. (The yellow shaded parts of the figure show results from a single model as illustration.) The multi-model relative standard deviation (Fig. 2, upper right) rarely goes below 50%. We note that it is lowest over high anthropogenic emission regions, where emissions were identical for all models. However, the relative standard deviation is very high over dust-emission regions, as dust emissions were not specified in the AeroCom exercise, but were estimated by each model based on meteorology for the same year. Also, for most models, a single dust “type” was assumed. One clear example of the implication of this assumption is the high absorption seen over the Bodélé depression (Chad, Central Africa), which is high in dust loading, but where the composition is mainly non-absorbing diatoms [106].

Most of the AeroCom models have undergone some degree of validation against observations, and the multi-model median holds up well when broadly evaluated against recent satellite remote sensing products (e.g., [98], see further discussion below). However, such an array of assumptions goes into these model calculations that it is difficult to assess the value of the (weak) multi-model, global-average constraint provided (0.0042 ± 0.0019). Consistency among models can arise due to common assumptions and/or tuning (see, e.g., [107]).

Another path towards model-based constraints is to combine information from multiple satellite retrievals with the full spatial coverage of a single numerical model, as done in the Monitoring Atmospheric Composition and Climate (MACC) project [108]. Here, model simulations are assimilated with MODIS retreivals of AOD (550 nm) and AERONET information on SSA, to derive AAOD. The use of assimilation combines global aerosol modeling and observations, and adds an additional layer of information relative to the earlier, purely observational, methods combining satellite and AERONET data [109, 110]. MACC estimates a global, total AAOD of 0.008 ± 0.002 (i.e., almost twice that of AeroCom Phase II), mainly from anthropogenic sources (which include all biomass burning emissions) (0.007 ± 0.001), with the remainder being ascribed to mineral dust (0.001 ± 0.001).

## Observational Advances and Perspectives

In the following, we discuss recent developments and known issues for ground and space-based remote sensing, and in situ measurements via regional flight campaigns and long-term surface monitoring sites. Presently, there is no practical way to provide global, self-consistent measurements from a single source that sufficiently constrains aerosol absorption for global modeling purposes. Still, satellite, aircraft, and surface station information can be used to validate or constrain global models, and therefore provide key, underlying constraints on climate scenarios and predictions.

Column aerosol absorption (i.e., AAOD) can, in principle, be retrieved from ground and space-based remote sensing platforms, though with uncertainties that limit their direct application for detailed climate forcing calculations (e.g., [111]). New instrumentation is in development that will provide a better understanding of the physical processes and hence the means to parameterize absorbing aerosol, but existing observational networks and datasets also provide crucial information. In situ observations can provide the most accurate observational constraints on aerosol spectral absorption and, depending on the instrument, such measurements likely meet the first-order requirements for climate forcing calculations [9, 63]. In situ measurements from aircraft have limited spatial and temporal coverage, whereas in situ measurements from long-term surface sites can provide the temporal coverage and hence climatology, but again spatial coverage is limited, and transported aerosol might not be sampled adequately by in situ surface stations.

### Remote Sensing by Ground Stations

Sun photometers at AERONET sites [112] provide AAOD retrievals at up to 600 locations around the globe, around 30% of which are in urban locations. As a long-running measurement network, AERONET provides invaluable climatological aerosol information; however, only a very few sites have operated consistently for the full range of about 20 years. Proper care must therefore be taken when selecting sites for climatological and/or trend absorption analyses. As with all passive ground-based technologies, AERONET can only observe total column properties. The AERONET algorithm also retrieves the aerosol complex refractive index, but it assumes it to be the same for all particle sizes in the atmospheric column [113]. Additionally, Level 2 absorption products such as AAOD require solar zenith angle > 50° and AOD > 0.4 at 440 nm. As AOD > 0.4 is uncommon ([114], their Fig. 1; the global average AOD at 440 nm is not far above 0.14), this requirement means that Level 2 AAOD information is limited, and skewed towards conditions of high aerosol loading.

As the largest ground-based network capable of constraining column AAOD, AERONET has recently been used to provide constraints on models [115], to estimate BC emission inventories [116], and to scale estimates of radiative forcing [2, 49]. Russell et al. [117] recently showed how a cluster analysis of AERONET particle property retrievals can be combined with global-scale, multi-angle, multi-spectral, polarized measurements from the POLDER (Polarization and Directionality of the Earth’s Reflectances) satellite instrument, to classify observations in terms of aerosol types, providing qualitative constraints on aerosol properties, including AAOD.

Schuster et al. [20] use the AERONET imaginary refractive index retrievals and published refractive indices to infer the relative abundance of BC, BrC, hematite, and goethite, using an end-member mixing approach. They utilized AERONET data at biomass burning sites and dust-dominated sites to illustrate that an imaginary index of *k* = 0.0042 at the 675–1020-nm wavelengths robustly separates “pure” carbonaceous from “pure” dust aerosols. By further assuming that the spectral dependence of the AERONET imaginary refractive indices are associated with BrC or free iron, and that most BrC resides in the fine mode while most free iron resides in the coarse mode, they were able to retrieve regional and seasonal column loadings of absorbing aerosols that are consistent with expectations. They estimate an uncertainty of about 50% or better for BC and 100% or better for free iron.

A recurring question in recent literature has been what requirement should be set on total AOD for AERONET to give a good AAOD retrieval. As input to this, AERONET has been extensively compared with various types of airborne in situ measurements. These were recently summarized in Andrews et al. [118]. See Supplementary Fig. 2, left panel, adapted from data used in that study. The authors compare results from two US continental AERONET sites with in situ profiles from aircraft observations, with emphasis on low aerosol loading conditions. They confirm a previously reported tendency for AERONET inversions to overestimate absorption at low AOD values, suggesting a bias in either the retrievals or the in situ techniques. Previously, Kahn et al. [119], in a similar analysis comparing AERONET with MISR satellite data, attributed underestimates in AERONET SSA at least partly to methodological differences in measurements of AERONET direct-sun extinction and sky scan scattering quantities. These points further suggest caution in using AERONET to scale global model results, and brings into question the assumption that AERONET SSA values retrieved at high and low AOD conditions can be used to obtain AAOD at low AOD conditions (e.g., [98, 120]). Thus AERONET SSA may not be representative of all loading conditions and/or seasons.

A further issue to note is vast differences in sampling size between most global models, at resolutions of 1° x 1° or coarser, and AERONET point measurements. Using a high resolution emission inventory for Asia, a nested climate model and downscaling techniques, Wang et al. [49] recently explored the impact of model resolution on model-AERONET bias and, subsequently, radiative forcing of BC. The analysis was made at 900 nm, where BrC was assumed not to contribute to absorption. Contributions from dust were estimated by assuming a set of spectrally dependent optical parameters. They found significant reductions in bias when using high-resolution modeling and emissions, and a reduction in predicted RF when using methods similar to previous studies. This is not surprising, as aerosol abundances are known to vary on small spatial scales compared to typical global-model resolution. It does, however, strongly indicate a need for higher resolution approaches to aerosol modeling if climate impacts are to be estimated with confidence.

### Remote Sensing by Satellites

Several space-borne remote sensing instruments are currently capable of constraining aerosol absorption, at least qualitatively. Here, we discuss recent evaluations of MISR, OMI, and PARASOL retrievals.

The Earth Observing System (EOS) MISR flies aboard the NASA EOS’s Terra satellite. It provides categorical constraints on particle size, shape, and absorption properties, distinguishing about 3–5 bins in particle size, 2–4 bins in SSA, and spherical vs. randomly oriented non-spherical particle shape under good but not necessarily ideal retrieval conditions [79, 121]. From multi-angle, multi-spectral remote sensing, aerosol type retrievals are much more sensitive to retrieval conditions than AOD. The MISR Standard aerosol retrieval algorithm runs automatically on the (approximately once-weekly) global dataset, with a climatology of 74 candidate aerosol mixture optical models, providing about a dozen aerosol-type distinctions where conditions warrant. The current MISR Standard algorithm (Version 22) tends to underestimate the occurrence of absorbing particles relative to ground (AERONET) measurements in situations where such particles are present, due in part to limitations in the algorithm particle climatology [122]. A more recent Research Algorithm, allowing many more aerosol component options, hundreds of candidate mixtures, empirical calibration refinement, and advanced surface modeling, can retrieve more information, particularly about particle absorption, and under a broader range of retrieval conditions, but only for individual case studies due to practical considerations [123, 124].

When many aerosol mixtures pass the MISR algorithm acceptance criteria, as frequently occurs when the AOD falls below about 0.15 or 0.2 [66, 111], there might be too little information in the observed MISR radiances to constrain aerosol type. Aerosol transport models, however, identify aerosol properties based on downwind advection from specified sources, so AOD is generally not a limiting factor. Li et al. [125] used model information to refine MISR aerosol microphysical property retrievals in conditions where many mixtures passed. They were able to trace remaining differences between the model, the constrained retrieval, and ground truth primarily to underestimations of AOD and AAE by the model in polluted regions, and missing aerosol types in the MISR product.

Another orbital platform frequently used to constrain aerosol light absorption is the OMI aboard the NASA EOS Aura satellite. Providing information mainly in the ultraviolet, it is able to retrieve AOD and AAOD at 388 nm. The retrieval interprets aerosol absorption of upwelling, Rayleigh-scattered light from below and depends in part on having good constraints on the aerosol vertical distribution. The sensitivity of this approach improves for elevated vs. near-surface aerosol. Recently, OMI has been used to help validate the MERRA-2 (and the earlier MERRAero) re-analyses based on the GEOS-5 model [126, 127]. In general, the retrieval and reanalysis are in good agreement. However, a general overestimate by MERRA2 over land was observed. In a set of regional analyses, they focus on the Sahara (for dust), Africa and South America (for biomass burning aerosols), and Asia (anthropogenic mix). Dust AAOD reanalysis was improved by implementing recent updates to optical properties [128], whereas observed mismatches in the biomass burning regions were attributed to insufficient treatment of absorbing organic carbon (i.e., BrC). Over Asia, much of which is dominated by anthropogenic emissions, they point to emission inventories as a main source of uncertainty. However, possible impacts of aerosol absorption enhancement by rapid coating (the lensing effect mentioned previously) in high pollution environments [41] were not systematically discussed. In another recent study, Zhang et al. [129] use OMI AAOD to constrain BC abundances and emissions over Asia with the adjoint of the GEOS-Chem model. Using an optimization technique, they were able to significantly reduce model biases against AERONET and in situ ground truth at urban sites. Their results are similar to those of Wang et al. [49], indicating that greater regional specificity in emission inventories and additional constraints from space-based instruments can resolve a large fraction of the present uncertainty in BC emissions and concentrations.

The ESA PARASOL instrument measures polarization along with multi-angle, multi-spectral observations. The GRASP algorithm (Generalized Retrieval of Aerosol and Surface Properties) aims at gleaning information about absorbing aerosols from these data [130, 131]. Early work with this algorithm shows considerable promise in constraining particle size distributions and indices of refraction over a range of conditions. In particular, the added polarization information helps constrain both particle size and real refractive index, which contribute to the retrieval of particle absorption. As applied to the POLDER instruments aboard PARASOL, sensitivity to coarse-mode particles is limited by a lack of wavelengths longer than 910 nm, and relatively coarse spatial resolution (6 km at nadir) complicates interpretation of retrievals where the surface or aerosol vary on kilometer scales. Lacagnina et al. [98] performed a thorough evaluation of PARASOL Standard Product AAOD versus AeroCom models, AERONET ground stations, and OMI, but only for ocean regions. They found that the ground and satellite remote sensing data compared well for AOD and AAOD (and SSA). The AeroCom models compared well against the remote sensing data for AOD, but the models produced much lower values for AAOD than the remote sensing data.

One critical issue for constraining the total climate effect of absorbing aerosols is their impact when located above clouds. In such cases, the high underlying albedo will enhance shortwave absorption, but both satellite and ground-based remote sensing have problems detecting the aerosol layer. Recently, improvements have been made by several satellite teams, leading to better constraints on above-cloud absorption. One method uses total and polarized radiances measured by POLDER, and has been shown to be efficient for detecting aerosols above clouds over the southeast Atlantic Ocean, Siberian biomass burning, and Saharan dust above clouds off the northwest coast of Africa [132]. For example, Peers et al. [133] recently compared the absorbing aerosols above clouds off the southwest coast of Southern Africa from the POLDER retrivals with several AeroCom models, and found that all models have lower AAOD above clouds compared to POLDER. The lower AAOD in models was primarily ascribed to lower AOD above clouds, but for those models showing reasonable AOD above clouds, the SSA was higher than in POLDER. Another method uses near-UV observations from OMI, simultaneously deriving the optical depth of the aerosol layer and the underlying cloud. This method has been tested with good results over the southern Atlantic Ocean [134]. Chand et al. [135] present a “color ratio” method, applied to CALIPSO data, to detect fine-mode, generally absorbing anthropogenic aerosol over cloud. Several groups expanded on this idea, using MODIS multi-spectral observations to simultaneously derive aerosol and underlying cloud optical thickness [136–139]. Yet another recent development is the use of geostationary satellite data from the Spinning Enhanced Visible and Infrared Imager (SEVIRI) in conjunction with A-Train [140]. Although no full constraint on above-cloud aerosol absorption yet exists, rapid progress is being made in this crucial area.

### In Situ Surface Stations

Unlike remote-sensing retrievals, surface in situ measurements are not limited to daytime or cloud-free conditions, thus high temporal-resolution patterns can be studied. Additionally, measurements of absorption can be made at quite low near-surface loading conditions. Several networks are in operation, including IMPROVE, GAW, and the Environmental Protection Agency’s STN (Speciation and Transport Network). One drawback of surface in situ networks is their limited spatial coverage—although the US and Europe support multiple surface networks making BC and/or aerosol absorption measurements with varying coverage density, other regions are much more sparsely represented.

Although measurements from surface in situ networks have been and continue to be used to evaluate model simulations of BC and dust (e.g., [101, 141–146]), one enduring issue for the comparison to be valid is ensuring that the measured quantity is the same as the modeled quantity (e.g., [9, 63]). The continuous nature of surface measurements means they can be used for trends studies (e.g., [101, 147]), climatologies in various regions (e.g., [34, 142]), and investigating inter-annual variability (e.g., [148]).

### In Situ Aircraft Measurements

Fully constraining the vertical profiles of aerosol abundances, and hence absorption, should ideally be done in situ, using instruments in aircraft, balloons or, possibly, drones [149]. Recently, wider availability of high quality instrumentation such as the SP2 have led to greatly improved measurements of vertically resolved concentrations, both from campaigns targeted at specific processes, regions, and/or seasons (e.g., A-FORCE [150], CLARIFY, SAMBBA [151]), and near-annual-mean coverage in remote regions (HIPPO [45], ATom; https://espo.nasa.gov/atom). However, flight information will always be limited in spatial and temporal coverage, relative to the wider view of satellites, and the continuous operation of some ground stations. Sampling issues therefore quickly arise when using aircraft data to constrain or validate models, as plumes or layers may be missed by the flights, while also being below the resolution of the models [152].

Above, we have discussed how dedicated flights were used to validate two particular AERONET sites [118], and how recent SP2 measurements indicate a significant loading of BrC at high altitudes over the continental USA [70]. Several groups also use flight information to constrain aerosol optical properties in a given regions. E.g. Lan et al. [40] find, from a flight campaign in an urban South China atmosphere, MAC of BC at 532 nm averaging 6.5 m^2^ g^−1^. A further use for aircraft measurements is to aid in constraining the radiative contribution of aerosol above clouds, as discussed above.

Even if it is sparse, the flight information available could be better utilized for constraining both models and retrievals. A first step is ensuring easy availability of consistent datasets. Here, recent initiatives such as the GASSP database [153] should be of great use in the future. Also, there is great potential in more systematic deployment of aircraft measurements, to constrain the average optical properties of aerosols in a given region and season. We discuss this further below.

As alluded to in the discussion of in situ BC instruments above, one issue that affects both surface and airborne in situ measurements of absorption is relating them to the ambient reality. In situ measurements have a tendency to change the sampling conditions (e.g., T, RH) from ambient. Some work has been done to couple SP2 with other systems to assess the hygroscopicity of absorbing aerosol (e.g., [154, 155]) which may be useful for adjusting absorption measurements to ambient humidity conditions. Another issue is the possible volatilization of condensed material (thus possibly changing the lensing effect) during sampling, particularly if heating is used to bring the sample air down to a desired measurement humidity. Switching to diffusion driers or dry air dilution systems may minimize this potential volatilization effect for in situ measurements.

## A Roadmap Towards Improved Constraints on Aerosol Absorption

In this review, we have defined a constraint as an agreement between observational and theoretical/model-based estimates of aerosol absorption, combined with an understanding of why the two are similar. At present, the field cannot provide such constraints. However, there is rapid progress on both fronts, and an encouraging increase in communication between the observational and modeling communities. In Table 1, we suggest a roadmap towards improved constraints, that includes model development, implementation of additional observational capabilities, and—crucially—increased adoption of common terms and definitions.

In summary, global and even regional-scale mapping of aerosol absorption remains challenging. No single source, models, in situ measurements, or satellite observations alone appears capable of providing the needed information to constrain absorption at the accuracy required for climate forcing applications, with adequate spatial and temporal coverage. Yet, taken together, this goal may still be achievable. Satellites offer frequent, global coverage and can map aerosol air mass types qualitatively, and with better constraints, under a wider range of observing conditions, when multi-angle, polarization data are acquired over a spectral range covering the near-UV to the near-IR. Details of particle absorption are best obtained from systematic, in situ measurements within the major aerosol air mass types. Although surface-based in situ measurements of aerosol absorption covering a range of air mass type exist due to the efforts of multiple monitoring networks, above-ground sampling is needed to adequately characterize transported aerosol types, and vertical profiles of in situ aerosol properties would better serve our ability to tie together satellite, remote sensing, and modeled absorption. Although such data are currently lacking in most cases, a climatological subset of key aerosol optical measurements has been acquired systematically within a single geographical region/altitude [156, 157], and a concept for comprehensive, global measurements of this type has recently been presented [158]. Once an extensive database of detailed particle optical and microphysical properties is acquired in situ, including PDFs of particle hygroscopicity, mass extinction efficiency (needed to translate between aerosol optical depth retrieved from remote sensing and aerosol mass book keeping in models), and aerosol spectral absorption, these can be associated with the aerosol air mass types mapped from space [111]. Models can contribute to this overall picture by helping constrain the results where the satellite aerosol type retrievals are ambiguous (e.g., [125]), or are lacking, e.g., due to cloud cover, and the model can then in turn be constrained and/or validated by the aggregated observations.

Surface-based in situ measurements currently provide an underutilized dataset for evaluating and constraining modeled absorption in the boundary layer. If deployed at existing, long-term surface sites, the state of the art instruments mentioned above could go a long way towards resolving uncertainties in climatological physical properties. This, in turn, could aid in interpreting the existing long-term monitoring data (e.g., PSAP measurements) in light of the issues summarized in this review.

Further key areas of great present interest are constraints on the vertical distribution of absorbing aerosol, and the impacts of absorbing aerosol above cloud. Vertical distributions may be constrained in models through a combination of near-source plume-heights, as determined, e.g., from multi-angle imaging, downwind layer profiles, retrieved, e.g., from space-based lidar, and the understanding that aerosol tends to concentrate in the boundary layer or in layers of relative stability in the free troposphere [159]. Such work has been suggested within the framework of the established AeroCom/AeroSAT collaboration, and may provide very relevant constraints in the future. Aerosol above cloud are presently being evaluated both from models and remote sensing (the OMI and MODIS teams in particular), and is a focus of the current ORACLES field campaign, but is at present still a major source of uncertainty for the total radiative forcing exerted by aerosols.

In conclusion, although progress is rapid, there is still need for much additional work in characterizing aerosol light absorption in the atmosphere, and its effects on radiative forcing and – ultimately – the climate.

## Electronic Supplementary Material

ESM 1 (DOCX 213 kb)
