# Supplementary material for: Aerosol Absorption: Progress Towards Global and Regional Constraints
Source: Curr Clim Change Rep. 2018 Apr 3;4(2):65–83. doi: 10.1007/s40641-018-0091-4 (PMC6448288; doi:10.1007/s40641-018-0091-4)
Supplement: Supplementary file 1 — (DOCX 213 kb) [file 40641_2018_91_MOESM1_ESM.docx]

Aerosol absorption: Progress towards global and regional constraints

# Supplementary Materials

## Supplementary Tables

Supplementary Table 1: Summary of issues broadly discussed in recent literature, and a selection of key publications.

| Physical properties and modelling | Current topics / issues | Recent key papers (selected) |
| --- | --- | --- |
| BC | Absorption enhancement during ageing, emission inventories, residence time, vertical concentration profiles | ^1-8^, and Supplementary Table 2. |
| BrC | Composition, wavelength dependence, lensing, absorption decay over time | ^9-16^ |
| Dust | Modeled source terms, size distributions, composition and assumptions on shape. | ^17-20^ |
| Model-based constraints | Optical properties, model process differences, assimilation | ^21-23^ |
| Remote sensing and in-situ measurements | Current topics / issues | Recent key papers (selected) |
| Remote sensing, ground stations | AERONET AAOD at AOD<0.4, representativeness of sites, retrieval assumptions, separation of species | ^24-29^ |
| Remote sensing, satellites | Separation of species, retrieval assumptions, aerosol above clouds | ^22,30-37^ |
| In-situ, surface stations | Limited spatial coverage, correspondence of measurements to model assumptions | ^38-42^ |
| In-situ, aircraft measurements | Limited spatial and temporal coverage | ^7,13,20,24,43^ |

Supplementary table 2: Overview of enhancement factors from a number of studies. ε indicates absorption enhancement factors calculated for freshly emitted BC, relative to pure uncoated BC. “Pure” refers to uncoated and collapsed BC, “Fresh” refers to freshly emitted BC, while “Aged” refers to aged BC that has become coated.

|  | E_abs,fresh_ | E_abs,aged_ | E_abs,total_ | λ (nm) |
| --- | --- | --- | --- | --- |
|  | Pure 🡪 fresh | Fresh 🡪 aged | Pure 🡪 aged |  |
| Bond and Bergstrøm (2006) ^44^ | 1.5 | 1.5 | (2.3) |  |
| Cappa et al. (2012) ^45^ | 1.06 | 1.2 |  | 532 |
| Cui et al. (2016) ^46^ | 1.4 | 1.7 | 3 | 678 |
| Peng et al. (2016) ^2^ |  |  | 2.4 | 532 |
| Liu et al. (2015) ^47^ | 1.1 | 1.4 |  | 781 |
| Healy et al. (2015) ^48^ | 1.0 |  |  | 781 |
| Nakayama et al. (2014) ^49^ | 1.1 |  |  | 781 |
| Sinha et al. (2017) ^50^ |  |  | 1.44 | 565 |
| Lan et al. (2013) ^43^ | 1.07 |  |  | 532 |
| Liu et al. (2017) ^6^ |  | 1.1-1.6 |  | 405, 532, 781 |

## Supplementary Figures


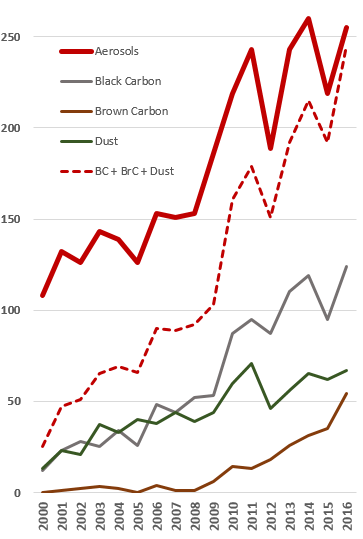


Supplementary Figure 1: Number of publications per year for aerosol absorption, and for the three main absorbing species. Listings taken from ISI Web of Knowledge, category Meteorology and Atmospheric Sciences. Keyword “absorption” was required for all papers, in addition to the terms in the legend. The dashed line shows the sum of black carbon, brown carbon and dust absorption publications.


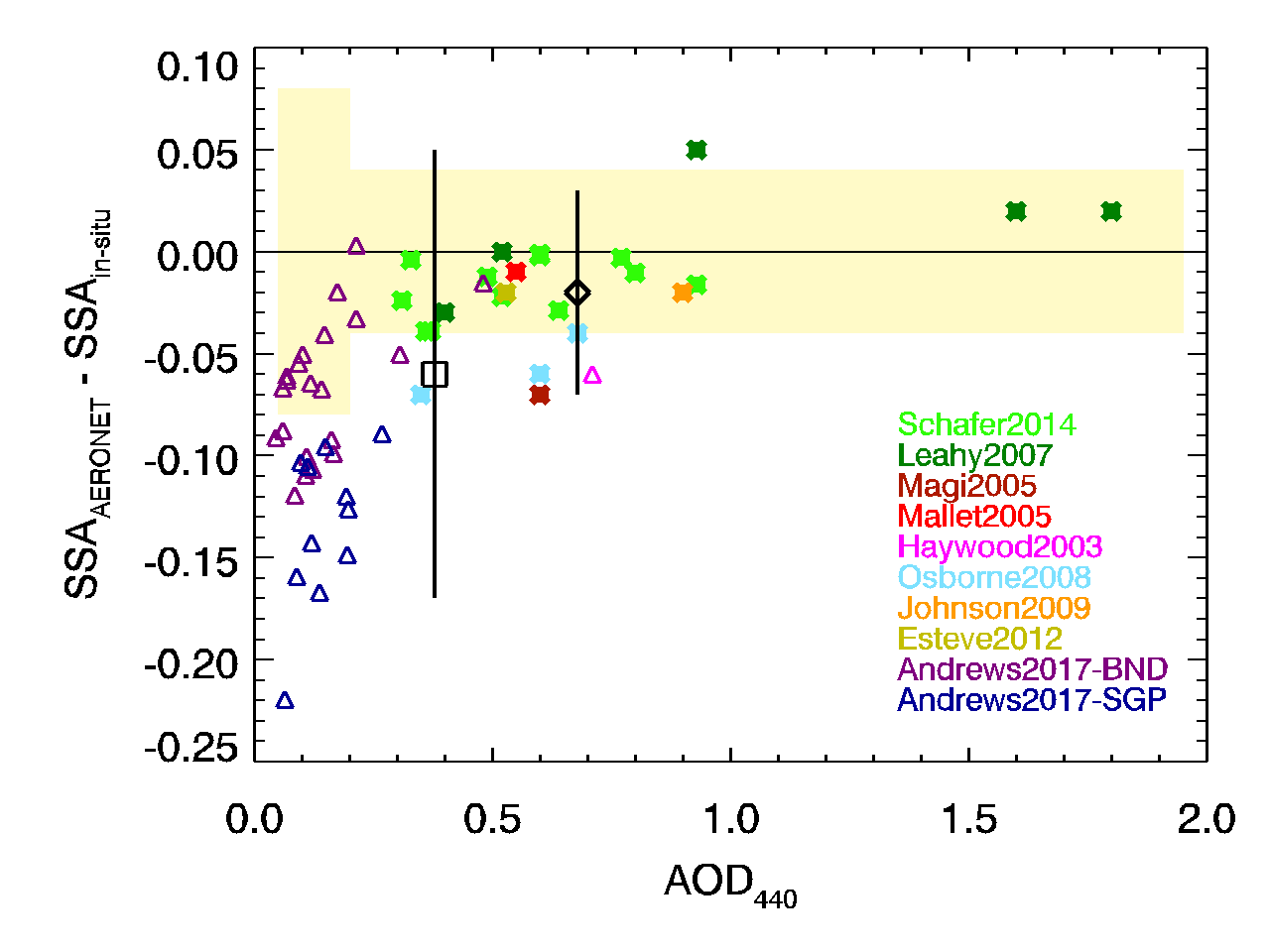

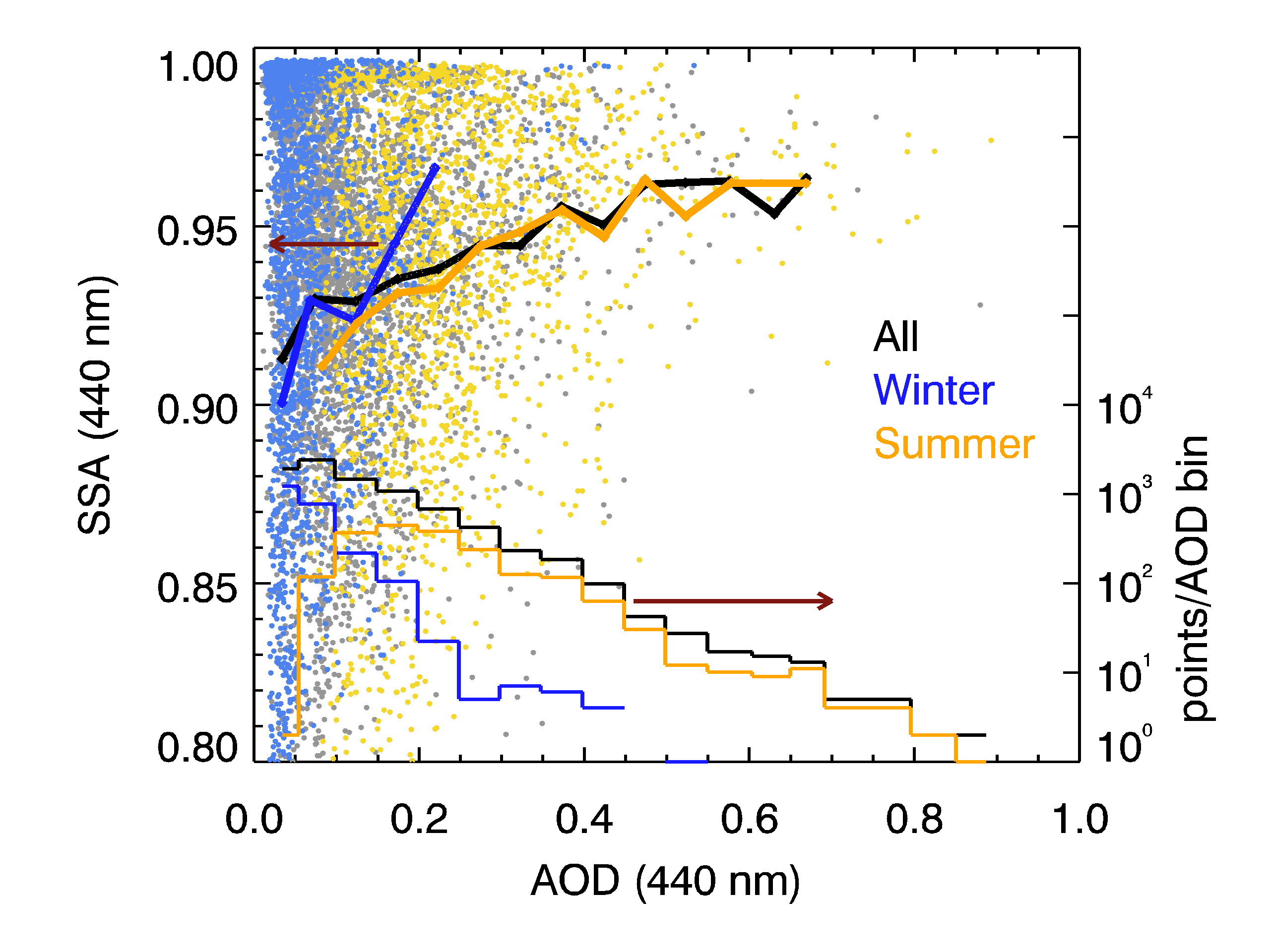


Supplementary Figure 2: Relationships between aerosol loading and SSA. Left panel: Differences in SSA obtained from multiple airborne, *in-*situ vertical profiling campaigns with SSA obtained from co-located, simultaneous AERONET retrievals as a function of AOD. Open symbols are for 440 nm SSA difference; filled symbols are for 550 nm SSA difference. Shading indicates combined uncertainty of AERONET SSA values as function of AOD, as reported in Table 4 of ^51^, and uncertainty in the in-situ SSA. (Data from ^24^, their Figure 7). Right panel: Relationship between SSA and AOD at the Southern Great Plains (SGP) AERONET site as a function of season for time period 1994-2016. Symbols represent individual retrievals for Level 2 data (constraints on SSA retrievals (e.g., AOD440>0.4) were ignored); thick lines in top half of plot represent medians of SSA and AOD data (left axis); thin stair step lines in lower half of plot represent number of points in each 0.05 AOD bin (right axis). The dark blue triangles in Fig. 4a result from comparing vertically integrated in-situ measurements during profile flights over the SGP site with concurrent AERONET retrievals from the AERONET dataset depicted in Fig. 4b.

# References

1 Liu, C., Chul, C. E. & Yin, Y. The Absorption Ångström Exponent of black carbon:

from numerical aspects. *Atmospheric Chemistry and Physics Discussions*, 1-30, doi:10.5194/acp-2017-836 (2017).

2 Peng, J. *et al.* Markedly enhanced absorption and direct radiative forcing of black carbon under polluted urban environments. *Proceedings of the National Academy of Sciences* **113**, 4266-4271, doi:10.1073/pnas.1602310113 (2016).

3 Boucher, O. *et al.* Jury is still out on the radiative forcing by black carbon. *Proceedings of the National Academy of Sciences* **113**, E5092-E5093, doi:10.1073/pnas.1607005113 (2016).

4 Stjern, C. W. *et al.* Rapid adjustments cause weak surface temperature response to increased black carbon concentrations. *Journal of Geophysical Research* (2017).

5 Fierce, L., Bond, T. C., Bauer, S. E., Mena, F. & Riemer, N. Black carbon absorption at the global scale is affected by particle-scale diversity in composition. *Nature communications* **7**, 12361, doi:10.1038/ncomms12361 (2016).

6 Liu, D. *et al.* Black-carbon absorption enhancement in the atmosphere determined by particle mixing state. *Nature Geosci* **10**, 184-188, doi:10.1038/ngeo2901 (2017).

7 Schwarz, J. P. *et al.* Aircraft measurements of black carbon vertical profiles show upper tropospheric variability and stability. *Geophys Res Lett*, doi:10.1002/2016GL071241 (2017).

8 Wang, R. *et al.* Estimation of global black carbon direct radiative forcing and its uncertainty constrained by observations. *Journal of Geophysical Research: Atmospheres* **121**, 5948-5971, doi:10.1002/2015JD024326 (2016).

9 Pokhrel, R. P. *et al.* Relative importance of black carbon, brown carbon, and absorption enhancement from clear coatings in biomass burning emissions. *Atmos. Chem. Phys.* **17**, 5063-5078, doi:10.5194/acp-17-5063-2017 (2017).

10 Feng, Y., Ramanathan, V. & Kotamarthi, V. R. Brown carbon: a significant atmospheric absorber of solar radiation? *Atmos. Chem. Phys.* **13**, 8607-8621, doi:10.5194/acp-13-8607-2013 (2013).

11 Saleh, R. *et al.* Contribution of brown carbon and lensing to the direct radiative effect of carbonaceous aerosols from biomass and biofuel burning emissions. *Journal of Geophysical Research: Atmospheres* **120**, 10,285-210,296, doi:10.1002/2015JD023697 (2015).

12 Wang, Q. *et al.* Modeling investigation of light-absorbing aerosols in the Amazon Basin during the wet season. *Atmos. Chem. Phys.* **16**, 14775-14794, doi:10.5194/acp-16-14775-2016 (2016).

13 Zhang, Y. *et al.* Top-of-atmosphere radiative forcing affected by brown carbon in the upper troposphere. *Nature Geosci* **10**, 486-489, doi:10.1038/ngeo2960 (2017).

14 Wang, X. *et al.* Exploring the observational constraints on the simulation of brown carbon. *Atmospheric Chemistry and Physics Discussions*, 1-36, doi:10.5194/acp-2017-655 (2017).

15 Liu, J. *et al.* Brown carbon in the continental troposphere. *Geophys Res Lett* **41**, 2191-2195, doi:10.1002/2013gl058976 (2014).

16 Peterson, D. A. *et al.* Detection and Inventory of Intense Pyroconvection in Western North America using GOES-15 Daytime Infrared Data. *Journal of Applied Meteorology and Climatology* **56**, 471-493, doi:10.1175/jamc-d-16-0226.1 (2017).

17 Kok, J. F. *et al.* Smaller desert dust cooling effect estimated from analysis of dust size and abundance. *Nature Geosci* **10**, 274-278, doi:10.1038/ngeo2912 (2017).

18 Ridley, D. A., Heald, C. L., Kok, J. F. & Zhao, C. An observationally constrained estimate of global dust aerosol optical depth. *Atmos. Chem. Phys.* **16**, 15097-15117, doi:10.5194/acp-16-15097-2016 (2016).

19 Zhang, X. L., Wu, G. J., Zhang, C. L., Xu, T. L. & Zhou, Q. Q. What is the real role of iron oxides in the optical properties of dust aerosols? *Atmos. Chem. Phys.* **15**, 12159-12177, doi:10.5194/acp-15-12159-2015 (2015).

20 Di Biagio, C. *et al.* Global scale variability of the mineral dust long-wave refractive index: a new dataset of in situ measurements for climate modeling and remote sensing. *Atmos. Chem. Phys.* **17**, 1901-1929, doi:10.5194/acp-17-1901-2017 (2017).

21 Myhre, G. *et al.* Radiative forcing of the direct aerosol effect from AeroCom Phase II simulations. *Atmos Chem Phys* **13**, 1-25, doi:doi:10.5194/acp-13-1-2013 (2013).

22 Lacagnina, C. *et al.* Aerosol single-scattering albedo over the global oceans: Comparing PARASOL retrievals with AERONET, OMI, and AeroCom models estimates. *Journal of Geophysical Research: Atmospheres* **120**, 9814-9836, doi:10.1002/2015jd023501 (2015).

23 Bellouin, N., Quaas, J., Morcrette, J. J. & Boucher, O. Estimates of aerosol radiative forcing from the MACC re-analysis. *Atmos Chem Phys* **13**, 2045-2062, doi:DOI 10.5194/acp-13-2045-2013 (2013).

24 Andrews, E., Ogren, J. A., Kinne, S. & Samset, B. Comparison of AOD, AAOD and column single scattering albedo from AERONET retrievals and in situ profiling measurements. *Atmos Chem Phys* **17**, 6041-6072, doi:10.5194/acp-17-6041-2017 (2017).

25 Wang, X. *et al.* Exploiting simultaneous observational constraints on mass and absorption to estimate the global direct radiative forcing of black carbon and brown carbon. *Atmospheric Chemistry and Physics Discussions* **14**, 17527-17583, doi:10.5194/acpd-14-17527-2014 (2014).

26 Cohen, J. B. & Wang, C. Estimating global black carbon emissions using a top-down Kalman Filter approach. *Journal of Geophysical Research: Atmospheres* **119**, 307-323, doi:10.1002/2013JD019912 (2014).

27 Russell, P. B. *et al.* A multiparameter aerosol classification method and its application to retrievals from spaceborne polarimetry. *Journal of Geophysical Research: Atmospheres* **119**, 9838-9863, doi:10.1002/2013JD021411 (2014).

28 Schuster, G. L., Dubovik, O. & Arola, A. Remote sensing of soot carbon – Part 1: Distinguishing different absorbing aerosol species. *Atmos. Chem. Phys.* **16**, 1565-1585, doi:10.5194/acp-16-1565-2016 (2016).

29 Schuster, G. L., Dubovik, O., Arola, A., Eck, T. F. & Holben, B. N. Remote sensing of soot carbon – Part 2: Understanding the absorption Ångström exponent. *Atmos Chem Phys* **16**, 1587-1602, doi:10.5194/acp-16-1587-2016 (2016).

30 Kahn, R. A. & Gaitley, B. J. An analysis of global aerosol type as retrieved by MISR. *Journal of Geophysical Research: Atmospheres* **120**, 4248-4281, doi:10.1002/2015jd023322 (2015).

31 Limbacher, J. A. & Kahn, R. A. Updated MISR dark water research aerosol retrieval algorithm – Part 1: Coupled 1.1 km ocean surface chlorophyll a retrievals with empirical calibration corrections. *Atmos. Meas. Tech.* **10**, 1539-1555, doi:10.5194/amt-10-1539-2017 (2017).

32 Li, S., Kahn, R., Chin, M., Garay, M. J. & Liu, Y. Improving satellite-retrieved aerosol microphysical properties using GOCART data. *Atmos. Meas. Tech.* **8**, 1157-1171, doi:10.5194/amt-8-1157-2015 (2015).

33 Buchard, V. *et al.* The MERRA-2 Aerosol Reanalysis, 1980 Onward. Part II: Evaluation and Case Studies. *J Climate* **30**, 6851-6872, doi:10.1175/jcli-d-16-0613.1 (2017).

34 Colarco, P. R. *et al.* Impact of radiatively interactive dust aerosols in the NASA GEOS-5 climate model: Sensitivity to dust particle shape and refractive index. *Journal of Geophysical Research: Atmospheres* **119**, 753-786, doi:10.1002/2013jd020046 (2014).

35 Zhang, L. *et al.* Constraining black carbon aerosol over Asia using OMI aerosol absorption optical depth and the adjoint of GEOS-Chem. *Atmos Chem Phys* **15**, 10281-10308, doi:10.5194/acp-15-10281-2015 (2015).

36 Torres, B. *et al.* Advanced characterization of aerosol properties from measurements of spectral optical depth using the GRASP algorithm. *Atmos. Meas. Tech. Discuss.* **2016**, 1-47, doi:10.5194/amt-2016-334 (2016).

37 Peers, F. *et al.* Comparison of aerosol optical properties above clouds between POLDER and AeroCom models over the South East Atlantic Ocean during the fire season. *Geophys Res Lett* **43**, 3991-4000, doi:10.1002/2016gl068222 (2016).

38 Appel, K. W. *et al.* Evaluation of dust and trace metal estimates from the Community Multiscale Air Quality (CMAQ) model version 5.0. *Geosci. Model Dev.* **6**, 883-899, doi:10.5194/gmd-6-883-2013 (2013).

39 Eckhardt, S. *et al.* Current model capabilities for simulating black carbon and sulfate concentrations in the Arctic atmosphere: a multi-model evaluation using a comprehensive measurement data set. *Atmos. Chem. Phys.* **15**, 9413-9433, doi:10.5194/acp-15-9413-2015 (2015).

40 Chin, M. *et al.* Multi-decadal aerosol variations from 1980 to 2009: a perspective from observations and a global model. *Atmos Chem Phys* **14**, 3657-3690, doi:10.5194/acp-14-3657-2014 (2014).

41 Collaud Coen, M. *et al.* Aerosol decadal trends – Part 1: In-situ optical measurements at GAW and IMPROVE stations. *Atmos. Chem. Phys.* **13**, 869-894, doi:10.5194/acp-13-869-2013 (2013).

42 Sherman, J. P. *et al.* A multi-year study of lower tropospheric aerosol variability and systematic relationships from four North American regions. *Atmos. Chem. Phys.* **15**, 12487-12517, doi:10.5194/acp-15-12487-2015 (2015).

43 Lan, Z.-J. *et al.* Light absorption of black carbon aerosol and its enhancement by mixing state in an urban atmosphere in South China. *Atmos Environ* **69**, 118-123, doi:10.1016/j.atmosenv.2012.12.009 (2013).

44 Bond, T. C. & Bergstrom, R. W. Light Absorption by Carbonaceous Particles: An Investigative Review. *Aerosol Science and Technology* **40**, 27-67, doi:10.1080/02786820500421521 (2006).

45 Cappa, C. D. *et al.* Radiative Absorption Enhancements Due to the Mixing State of Atmospheric Black Carbon. *Science* **337**, 1078-1081, doi:10.1126/science.1223447 (2012).

46 Cui, X. *et al.* Radiative absorption enhancement from coatings on black carbon aerosols. *Sci Total Environ* **551**, 51-56, doi:<http://dx.doi.org/10.1016/j.scitotenv.2016.02.026> (2016).

47 Liu, S. *et al.* Enhanced light absorption by mixed source black and brown carbon particles in UK winter. *Nature Communications* **6**, 8435, doi:10.1038/ncomms9435

<http://www.nature.com/articles/ncomms9435#supplementary-information> (2015).

48 Healy, R. M. *et al.* Light-absorbing properties of ambient black carbon and brown carbon from fossil fuel and biomass burning sources. *Journal of Geophysical Research: Atmospheres* **120**, 6619-6633, doi:10.1002/2015JD023382 (2015).

49 Nakayama, T. *et al.* Properties of light-absorbing aerosols in the Nagoya urban area, Japan, in August 2011 and January 2012: Contributions of brown carbon and lensing effect. *Journal of Geophysical Research: Atmospheres* **119**, 12,721-712,739, doi:10.1002/2014JD021744 (2014).

50 Sinha, P. R. *et al.* Evaluation of ground-based black carbon measurements by filter-based photometers at two Arctic sites. *Journal of Geophysical Research: Atmospheres* **122**, 3544-3572, doi:10.1002/2016JD025843 (2017).

51 Dubovik, O. & King, M. D. A flexible inversion algorithm for retrieval of aerosol optical properties from Sun and sky radiance measurements. *Journal of Geophysical Research: Atmospheres* **105**, 20673-20696, doi:10.1029/2000JD900282 (2000).
